# What Do We Know Today about Long COVID? Nursing Care for a New Clinical Syndrome

**DOI:** 10.3390/ijerph19148642

**Published:** 2022-07-15

**Authors:** Rosa M. Cárdaba-García, Carlos Durantez-Fernández, Lucía Pérez Pérez, María Ángeles Barba-Pérez, Elena Olea

**Affiliations:** 1 Emergencies Management (SACYL), 40002 Segovia, Spain; rosamaria.cardaba@uva.es; 2 Nursing Department, Faculty of Nursing, University of Valladolid, 47005 Valladolid, Spain; lucia.perez@uva.es (L.P.P.); mariaangeles.barba@uva.es (M.Á.B.-P.); elena.olea@uva.es (E.O.); 3 Nursing Care Research (GICE), Faculty of Nursing, University of Valladolid, 47005 Valladolid, Spain; 4 Faculty of Health Sciences, University of Castilla-La Mancha, 45600 Talavera de la Reina, Spain; 5 Primary Care Management Valladolid West (SACYL), 47012 Valladolid, Spain; 6 University Clinical Hospital of Valladolid, 47003 Valladolid, Spain; 7 Unit of Excellence, Institute of Biology and Molecular Genetics (IBGM), University of Valladolid-CSIC, 47005 Valladolid, Spain

## 1. Long COVID: The Disease That Lasts

Persistent COVID, long COVID, long-effects, long-term effects or chronic COVID are all names of a new syndrome caused by a set of multi-organ symptoms that appear after having been infected with SARS-CoV-2 [1]. These symptoms occur 4 or even 12 weeks after the acute phase of COVID-19 disease and persist over time. These are not sequela or what is commonly referred to as post-COVID, which is due to severe acute SARS-CoV-2 disease and causes hospital admission and even intensive care. In long COVID, symptoms occur regardless of the severity of the disease in the acute phase. Furthermore, there is no cure period per se, as the symptoms do not disappear, but rather often occur in outbreaks. However, having long COVID is not incompatible with suffering from sequela related to having gone through the acute phase severely [2].

According to recent studies, it is estimated that about 10% of COVID-19 patients do not fully recover and develop persistent COVID after acute infection [3,4]. Some authors put this figure as high as 20% [5]. It is true that in the early stages of the pandemic, when the priority was to save as many lives as possible and in the face of very high mortality due to a new virus, this situation went unnoticed and was of little importance, but as the pandemic has been brought under control and health systems have stopped collapsing, this new clinical syndrome has become known. Patients suffering from it need a response to the symptoms they experience and to their needs arising from the disabilities generated by the virus [4]. As early as 2020, the WHO warned that this virus was not just killing people and that the long-term effects of COVID-19 should be taken into account by governments [6], and in December 2020 the National Institute for Health and Care Excellence (NICE) published guidelines on symptom management in long COVID patients [7].

It is not uncommon that, despite the recognition of this new syndrome by prestigious organizations worldwide, patients feel that there are innumerable barriers to accessing the healthcare system or to getting the response that they need. Additionally, there are difficulties in accessing disability benefits. The battle of patient groups, such as Long COVID ACTS, has been successful, because more and more scientific associations are issuing recommendations for the treatment of these patients [8,9].

## 2. Symptoms Accompanying Long COVID Syndrome

The variability of symptoms accompanying long COVID syndrome is very high; in this context, symptom maps have been produced that allow a comprehensive visualization of the incidence of symptoms. The symptom map from a national study in Spain found up to 201 symptoms related to long COVID [8]. In any case, it is necessary to remember that the symptomatology appears in the form of outbreaks and not in continuity [10].

Undoubtedly, the cardinal symptom is asthenia, which appears in almost all cases [11]. Other general symptoms include fever, chills, anorexia, and general malaise [12]. At the cardiac level, patients experience palpitations, orthostatic hypotension, hypertensive crises, syncope, and sinus bradycardia [13]. The respiratory system usually presents dyspnea, but dry cough and chest tightness may also occur. Coagulation may be affected by hematomas and acral ischemia. Dermatologically, the appearance of urticaria or skin rush and even alopecia is not uncommon. The digestive system may present abdominal pain, dyspepsia, pyrosis, flatulence, and diarrhea [14]. Neurologically, headaches, paresthesia, anosmia, memory deficits, instability, dizziness, and lack of concentration may occur. The ophthalmological apparatus may be altered in the form of diplopia, nystagmus, blurred vision, and dry eye [15]. The most common otolaryngology pathology is dysphagia and odynophagia, oral thrush, tinnitus and hypoacusis [16]. On a psychological level, anxiety, phobias, apathy, insomnia, and even obsessive compulsive disorders may appear [17].

## 3. What Measures Should Be Taken with a Long COVID Patient?

The situation of these patients is analogous to other patients with comorbidities, thus management should be similar. Initially, a therapeutic plan should be drawn up based on their needs, for which it is necessary to use scales to assess their physical, psychological, and social quality of life. If the assessment of the health of these people is comprehensive, the therapeutic regime must also be comprehensive.

At the beginning, at least the following assessment of the patient is necessary:

- Nutritional assessment, due to the situation of deficiency caused in some cases by acute infection. Weight, height, BMI, and total protein, albumin, ferritin, vitamin B12 and vitamin D should be measured in laboratory tests. Screening can be performed using the MUST tool [18].
- Sarcopenia assessment by loss of muscle mass. Physical tests to measure muscle strength and physical performance, such as the SARC-F scale, are used for this purpose [19].
- Dependency assessment, mainly in elderly or multi-pathological patients, by means of the Barthel scale [20].
- Emotional assessment, due to the psychological impact of the disease. There are countless scales for this, such as the EADG questionnaire [21].
- Social assessment, due to the impairment of social life associated with long COVID. The FSQ scale can be used to determine social participation [22].

## 4. Nursing Care in Long COVID Patients

Nursing in the 21st century is a profession with a scientific capacity and is its own professional field. The purpose of nursing is to provide for the patient if he or she is unable to respond autonomously to his or her own needs and to empower him or her to be self-sufficient and independent in his or her care, not only in terms of techniques, but also in terms of knowledge about their illness [23].

Furthermore, the nurse does not focus on the exclusively biological aspect of the patient, but takes into account, in a holistic way, his or her psychological and social sphere, and within this social sphere includes his or her primary circle of support, which is usually the family. The nurse therefore supports the health decisions of the person, family, and community [24].

Problems that are detected by nursing must be followed up in a planned way. It is not always possible to carry out the follow-up in the consultation room and it may be necessary to carry it out in the home environment, for which the nurse conducts the home consultation [25].

In addition, nurses should work with the caregivers of long COVID patients as a vulnerable group and support them in the informal care they provide, which is necessary for the health care system. Monitoring caregiver fatigue is an additional nursing function [26].

Setting up health education groups for long COVID patients helps to raise awareness of the syndrome and facilitates peer-to-peer contact, with proven multiple benefits for the patients [27].

In this type of patient, the figure of the nurse case manager who accompanies the patient and coordinates their care between care levels and between health and social health care is increasingly necessary. This figure avoids duplication, loss, and omission of consultations [28].

Exposing the existence of these problems to the community to prevent them from falling into oblivion or being stigmatized is a fundamental task, and to this end the nurse can contact the health agents in the community to make them known [29].

## 5. Conclusions

In conclusion, Long COVID affects 10–20% of the population that has had COVID 19 disease. The symptoms can be multiorgan and very diverse, affecting patients in the form of outbreaks. More and more Long COVID patient associations need more attention to their pathology.

There are different scales for nutritional assessment, loss of body mass, dependency and emotional, among others, that would help increase knowledge about persistent COVID and the needs of affected people.

Knowledge of the symptoms of these patients and their consequences for health, as well as the importance of the work of nurses in the care and management of these patients, is essential to understand Long COVID and improve the health and quality of life of these patients.
